# The regulatory effect of choice in *Situation Selection* reduces experiential, exocrine and respiratory arousal for negative emotional stimulations

**DOI:** 10.1038/s41598-017-12626-7

**Published:** 2017-10-03

**Authors:** Simon Thuillard, Elise S. Dan-Glauser

**Affiliations:** Institute of Psychology, University of Lausanne, Lausanne, Switzerland

## Abstract

*Situation selection* is a seldom studied emotion regulation strategy that entails choosing an upcoming emotional situation. Two mechanisms may drive its regulatory effect on emotional responses. One relates to the evaluation of the chosen option, people generally selecting the most positive. The other one implies that *having the choice* regarding the upcoming emotional situation is already regulatory, independently of what we choose. This research aimed at investigating this latter hypothesis. In a within-subject design, we compared emotional responses of 65 participants when they viewed negative and positive images they could select (use of *Situation selection*) vs. when they were imposed the *exact same images* (*Situation selection* not used). Results show that having the choice in negative contexts decreased negative experience, skin conductance, and respiration reactivity, while enhancing expressivity and cardiovascular reactivity. In positive contexts, choosing generally reinforced the image calming effect. Thus, contrary to other strategies that are efficient for negative but usually impair positive reactions (e.g., distraction), *Situation selection* may be used widely to reduce negative experience, while avoiding depletion of positive responses. This is particularly notable in emotion experience. Remarkably, these effects are not driven by the content of the situations, but by the act of choosing itself.

## Introduction

Emotion is a central aspect of life. It occurs over a relative short period of time^1,2^, and generally involves motivational stances^3,4^ and changes in three emotion response types: experience, expressivity, and physiological arousal^5–7^. Because of social display rules and individual preferences, a significant portion of emerging emotional episodes are regulated^8^. Emotion regulation refers to the processes involved in any attempt to modify emotional reaction unfolding, either by acting on the situation, the attention (or the meaning we give to it), or the responses that arise within us^9^. As a consequence, emotion regulation alters the trajectories of the unfolding emotion at the experiential, expressive, and physiological levels and, when effective, shapes the affective outcomes. The primary function of emotion regulation is the down-regulation of negative affect, and the enhancement of positive affect to increase well-being. Emotion regulation plays a crucial role in healthy adaptation^10,11^ and social functioning^12^; whereas difficulties in emotion regulation have been associated with pathologies such as substance dependencies^13^, or anxiety and mood disorders^14^. Functional emotion regulation is therefore crucial in promoting health and adaptation to the environment. Better identifying the mechanism and the specific impact of different emotion regulation types, or *strategies*, is thus of paramount importance to guide emotion regulation implementation in healthy individuals, as well as in patients.

One of the most influential model of emotion regulation is the Process Model of Emotion Regulation^15,16^, which presents five strategies used at different times along the emotion generative process: situation selection, situation modification, attentional deployment, reappraisal and suppression. *Reappraisal* (i.e., changing the meaning of an emotional situation) and *suppression* (i.e., modifying a behaviour, mainly the expressivity, to hide emotion manifestation) are two strategies that are often compared with one another. Past studies on the consequences of these two strategies helped  to better define why a particular strategy may be efficient in reducing negative emotional responses. Indeed, research suggests that *reappraisal* is a more adaptive strategy than *suppression* (see the meta-analysis by Webb and collaborators^17^). This more efficient functioning of *reappraisal* was explained by the fact that the earlier a strategy intervenes in the emotion generative process, the more chances it has to be efficient^18^. *Situation selection* should therefore be a particularly important strategy as it is the most antecedent one. *Situation selection* “involves taking actions that make it more (or less) likely that we will end up in a situation we expect will give rise to desirable (or undesirable) emotions”^19^ ^, p.11^ (see also^20^). S*ituation selection* thus involves selecting from different options the one to live^20^. By choosing to live or not a situation, individuals alter their future emotion unfolding, thus performing emotion regulation. *Situation selection* being a choice between options, it involves (a) an anticipation of affective consequences^21^, and (b) a careful weighting of the short vs. long-term consequences, or outcomes, of a particular option^9,19^.

One of the mechanisms through which *Situation selection* impacts emotion relates to the comparative evaluation of the available options. In general, people will choose the option that they evaluate as the most positive and/or beneficial to them. This choice can be driven by intrinsic features of the situation such as its valence, or by more extrinsic factors such as the chooser’s characteristics. Regarding the intrinsic features of the stimulus content, Sands & Isaacowitz^22^ showed that choices in a *Situation selection* procedure are guided by the information we can obtain about the arousal and valence levels of the upcoming situations, two long-identified dimensions of affect^23,24^. It is concluded that people generally like to engage in positive situations rather than negative ones and, if confronted to several positive situations, will choose the less arousing one. These may in part explain why *Situation selection* may have a positive impact on emotion, the chosen (more positive) situation intrinsically leading to less negative states^20^. Regarding extrinsic features of the stimulus that guide  the choice pattern, *Situation selection* has been for example suggested to be sensitive to cultural background, which encourages seeking emotional experiences that are desirable in a particular culture^25^. In a study by Bresin & Robinson^26^, authors operationalize *Situation selection* by contrasting positive and negative situations to choose from. The recorded choice patterns were shown to be related to the personality trait of agreeableness, showing more positive selection should this trait be high. Finally, a study by Rovenpor, Skogsberg, & Isaacowitz^21^ investigated patterns of choice when several choices of emotional material were given. Their study shows that what people choose and the number of minutes spent interacting with the given material are contingent upon the interaction of age and control beliefs. Globally, these results show that patterns of choice depend on who is choosing and what options are presented.

Beyond considering the available options based on arousal, valence and our personal features, we could make the hypothesis that there is a second mechanism by which *Situation selection* functions. This second mechanism deals with the effect of just ***having the choice***. Thus, independently of who is performing the choice, what choice is given, or what option is selected, emotional responses to two identical situations could be different if we have chosen the situation, or not. The possibility to perform choices is the cornerstone of freedom and human rights, and a crucial aspect of survival^27^. Since Seligman’s rationale^28,29^ that choice and control are fundamental to avoid learned helplessness, freedom of choices has been recognized as inherently rewarding^30^, and an important aspect of learning^31^, self-determination and well-being^32^. Coming back to emotion regulation, *Situation Selection* could thus have a double regulatory impact. First, as elaborated in the previous paragraph, by giving the opportunity to select less negative, or less arousing situations, which, in comparison to the neglected option, will lead to a better emotional outcome. Second, independently of the situation chosen, the fact of exerting control over the upcoming emotional situation is already regulatory *per se*. The impact of self-agency and sense of control on motivation has already been evoked in the literature^33^, but whether the act of choosing has a direct and immediate impact over emotion responses remains to be investigated.

The present study investigates what is the regulatory effect of just being given the choice. This study has been designed to experimentally evaluate how choosing a particular situation may impact the emotional reaction to it, and, this, independently of both the stimulus and the characteristics of a person performing the emotion regulation. This is achieved thanks to a highly-balanced stimulus presentation design to control for situations, coupled with a within-subject design, permitting to control inter-individual variability, a crucial aspect for psychophysiological data interpretation. Two conditions will be contrasted: a Chosen condition, in which participants perform *Situation selection*, i.e., choose the upcoming emotional situation, and an Imposed condition, in which participants do not have the choice on the situation they are about to see, i.e., they can’t perform *Situation selection*. Very importantly, and as detailed in the Method section, the images presented in each condition were matched to get rid of the stimuli content effect. By comparing Chosen and Imposed conditions while participants are seeing the exact same images, this design allows the extraction of the *unique* effect of the choice during *Situation selection*.

Given the velocity of emotional arousal and the importance of time in the emotional unfolding^34,35^, we examined the first eight seconds of participants’ emotional responses and include time as a factor. The three most immediate emotion response types highlighted by Gross’s model were targeted with (a) a continuous assessment of subjective emotion experience, (b) continuous facial electromyography for assessing expressivity, and (c) continuous measures of cardiovascular, respiratory, and somatic responses.

We hypothesized that selecting a particular situation would be efficient in decreasing negative and enhancing positive emotions, just by the act of choosing. The chosen condition (as compared to the imposed condition) was expected to have different response outcomes for each emotion parameter and for negative and positive contexts. Regulation strategies having shown to differentially impact the different emotion responses^36–42^, and emotional responses having dynamic and rapid emergence (particularly in reaction to emotional pictures^2,43–45^), we expect *Situation selection* impact to be particularly visible in the last few seconds of the viewing, when emotion responses become maximal. Regarding experience, we expected a decrease in negative emotion and enhanced positive experience. For expressivity, we expected decreased negative and enhanced positive expressivity. Regarding physiological arousal, we expected for both valences a decrease of the cardiovascular orienting effect (i.e., a decrease in heart rate and pulse activity) as found in similar paradigms for other strategies^36,37^, a decrease in skin conductance reactivity (particularly for negative viewing), a smaller increase in respiratory rate and smaller decrease in respiratory amplitude. Due to the scarcity of research measuring somatic activity other than for control purpose, we did not have reliable bases to define hypotheses for this measure.

## Methods

### Participants

A power analysis using a power of 0.8^46^, effect sizes based on previous studies with similar factors and measures, and an alpha of 0.05 for two tailed-tests, yielded a target sample size of 50. To be able to compensate for technical difficulties or signal artefacts, a sample size of 65 participants was targeted. To reach this goal, 72 participants were invited to the first session. Three of them were further excluded because of non-compliance with inclusion criteria and four further participants did not return for the second session. Sixty-five participants thus fully participated in our study (33 males and 32 females). Participants were either first year Psychology students participating for course credits (N = 45), or other discipline first year students participating for the equivalent of USD50 (N = 20). Participants were recruited through intervention in psychology course or ads displayed in university buildings. Study was briefly presented, without mention of emotion regulation as the focus. Participants’ ages ranged between 18.8 and 44.6 years, with a mean of 21.8 years (SD = 3.4 years). Inclusion criteria were age between 18 and 45 years old, no medication, and general good health. Regarding this latter point, participants were tested with the 12-Item Short-Form Health Survey SF-12^47^, and scored an average of 75.3% (SD = 12.3) of good health (100% being excellent health on every domain of the test). Since handedness may have an influence on emotion processing and physiological outputs^48,49^, all participants had to be right-handed. Their scores on the Edinburgh Handedness Inventory^50^, scoring -100 for totally left-handed and +100 for totally right-handed respondents, averaged 70.2 (SD = 20.9).

### Operationalization of *Situation Selection*

We contrasted two main conditions in a picture-viewing paradigm. The first was the counterpart of *Situation selection*, i.e., a condition, in which participants didn’t have the option to choose and were imposed to watch emotionally relevant negative and positive pictures (the Imposed condition). Despite participants could use self-regulated emotion regulation strategies, we consider this condition as *unregulated*, in contrast to the second condition, in which one specific strategy (*Situation selection*) was systematically used by the participants. In this second condition (the Chosen condition), we operationalized *Situation selection* by asking the participants to perform a choice between two options. Options were presented with words stating what kind of content was possible to be seen. Four negative and four positive contents were available (see the stimuli section below). Throughout the whole study, every choice was respected and the image the participant chose was presented right after the choice.

Whether chosen or imposed, pictures were shown with the same duration, size, at the same distance and with the same lighting. The particularity of the methodology is that each stimulus was shown twice to each participant: once in a chosen condition, and once in an imposed condition (in a randomized order to control for habituation). We could thus compare the emotional reactions *to the same emotional stimuli*, getting rid of the effect of the intrinsic emotional content of the presented stimuli. In other words, potential differences are not to be attributed to the fact that participants see less arousing or less emotional categories of images, but rather to one of the underlying mechanism behind *Situation selection*: the fact of having the choice.

### Stimuli

Eighty-seven pictures were selected from the Geneva Affective PicturE Database GAPED^51^. The GAPED gathers negative and positive stimuli that can be included in different content categories, each called with a label. This was particularly important to allow participants to perform *Situation selection* based on word descriptors of the upcoming situations. Pictures in the negative category consisted of four content types: spiders, snakes, animal mistreatment and human mistreatment. These words were those used as labels to offer the choice between categories in the Chosen condition. Pictures in the positive category also consisted of four content types: landscapes, human babies, mammals (generally offsprings), and sport (inspirational) pictures. Since examples of the latter content type are rare in the GAPED, we added nine pictures of sport/inspirational pictures from the International Affective Picture System IAPS^52^. Labels used to offer the choice for positive categories were “Landscape”, “Baby”, “Mammal”, and “Sport”. Of the final 96 pictures, 48 were negative and 48 were positive with 12 pictures of each content type.

### Measures

Emotion responses involve three main reactivity domain: emotion experience, expressivity, and physiological arousal^53–55^. In the present study, we wanted to tap into these different domains to have a comprehensive overview of emotion reactivity modulation by the choice component of *Situation Selection*.

#### Emotion experience

Participants used a rating slider for continuously reporting their emotion experience (Variable Assessment Transducer, Biopac Systems, Inc., Goleta, CA, USA) over the full duration of the picture presentations. The voltage output (0-9 V) was extracted as is.

#### Emotion-expressive behaviour

Expressivity was assessed using bipolar surface EMG electrodes were standard 4 mm Ag-AgCl sensors. Left *Corrugator Supercilii*, left *Zygomaticus Major*, and left *Orbicularis Oculi* were the three targeted regions. The corrugator region was targeted because of its reliable indication of negative expressivity^44,56^. Zygomatic region is generally used for measuring positive expressivity. However, contrary to Corrugator for negative expressivity, Zygomatic is a less direct measure of positive expressivity^56^. To compensate, this, but to keep this widely used measure, we decided to add an additional channel, targeting Orbiculari Oculi contractions, which are recognized to be a reliable readout (together with the zygomatic muscle) of real (Duchenne’s) smile^57^. Electrode placement followed recommendations by Fridlund and Cacioppo^58^. Skin was first gently rubbed with NuPrep® gel (Weaver and Cie). Excess gel was then removed with alcohol pads (Kendall Webcol® skin cleansing alcohol pads, Tyco Healthcare). Electrodes were filled with Signagel® (Parker Laboratories, Inc).

#### Autonomic and somatic responses

In order to tackle different systems involved in autonomic reactivity, measures focused on cardiovascular activity (1–3), exocrine activity (4), and respiratory activity (5). Somatic responses involved recording leg movements (6).

1. Electrocardiography (ECG): Three standard disposable pre-gelled Ag/AgCl electrodes were used for ECG recordings. One was placed approximately 5 cm below the lower rib on the left side of the abdomen. A second electrode was placed just under the right clavicle, along the mid-clavicular line. A third electrode, which functioned as a ground, was placed at the level of the xiphoid process.
2. Pulse: The variation of amplitude of the blood volume at the finger site was recorded with a photoplethysmography transducer from Biopac Systems (Goleta, CA, USA). It was clipped onto the extremity of the middle finger of the non-dominant hand. Pulse at the ear site was also recorded but due to scarcity of background literature using this measure, lack of trustable guidelines about analyses and interpretation, and absence of clear hypothesis about how this measure should be impacted by emotion emergence and regulation, these data were not analysed.
3. Skin temperature: Finger temperature was recorded with a temperature probe from Biopac Systems (Goleta, CA, USA), taped to the palmar surface of the extremity of the fourth finger of the non-dominant hand.
4. Electrodermal activity: Skin conductance level was recorded with two pre-gelled disposable Ag/AgCl sensors from Biopac Systems (Goleta, CA, USA). They were placed on the thenar and hypothenar eminences of the non-dominant hand palm.
5. Respiration: Thoracic and abdominal respiration recordings were gathered with two respiration belts from Biopac Systems (Goleta, CA, USA). The abdominal belt was placed around the waist just above the pants, whereas the thoracic belt was placed high on the chest just below the armpits.
6. Leg movements were recorded by placing a three-axial accelerometer above the ankle of the dominant leg.

All parameters (including rating, EMG, autonomic and somatic measures) were recorded and amplified with MP150 compatible modules from Biopac Systems (Goleta, CA, USA). All acquired channels were sampled at 1000 Hz.

### Procedure and Design

Participation was divided into two sessions. A questionnaire session, and the main testing phase including *Situation selection* testing.

#### Session 1: Questionnaires

In the first session, participants came into the lab and filled in some questionnaires on a computer (SF12, the Edinburgh Handedness Inventory, see *Participant* section, and other emotion-related questionnaires that served for another study, see Supplementary Note online for a full list). After the session, participants were invited to enlist for the second session of the experiment.

#### Session 2: *Situation selection* task

When arriving in the lab for the second session (about 10 days after the first session), participants were informed about the general ongoing of the procedure and prepared for the physiological recordings. Participants were then left alone, all instructions being presented on screen. Participants were told that we were interested in how people react while seeing different scenes. They were told that they were going to be shown different emotional pictures. Participants were then presented with the rating dial and explained that the major task of the study was to report their feeling by moving the cursor during the viewing of all pictures. At this stage, participants underwent a few training trials to get accustomed to the rating. They were then instructed about the emotion regulation task. Instruction was: “Sometimes in this session (in some blocks), you will have the opportunity to choose yourself, among two options, what image you would like to see. With the help of the arrows, select the image, then get back to the slider and concentrate on your feeling to report it with the cursor”. Participants again performed a few training trials (with images not presented in the main session) in which they chose between two proposed options and reported their feeling during the picture viewing.

**Figure 1:**
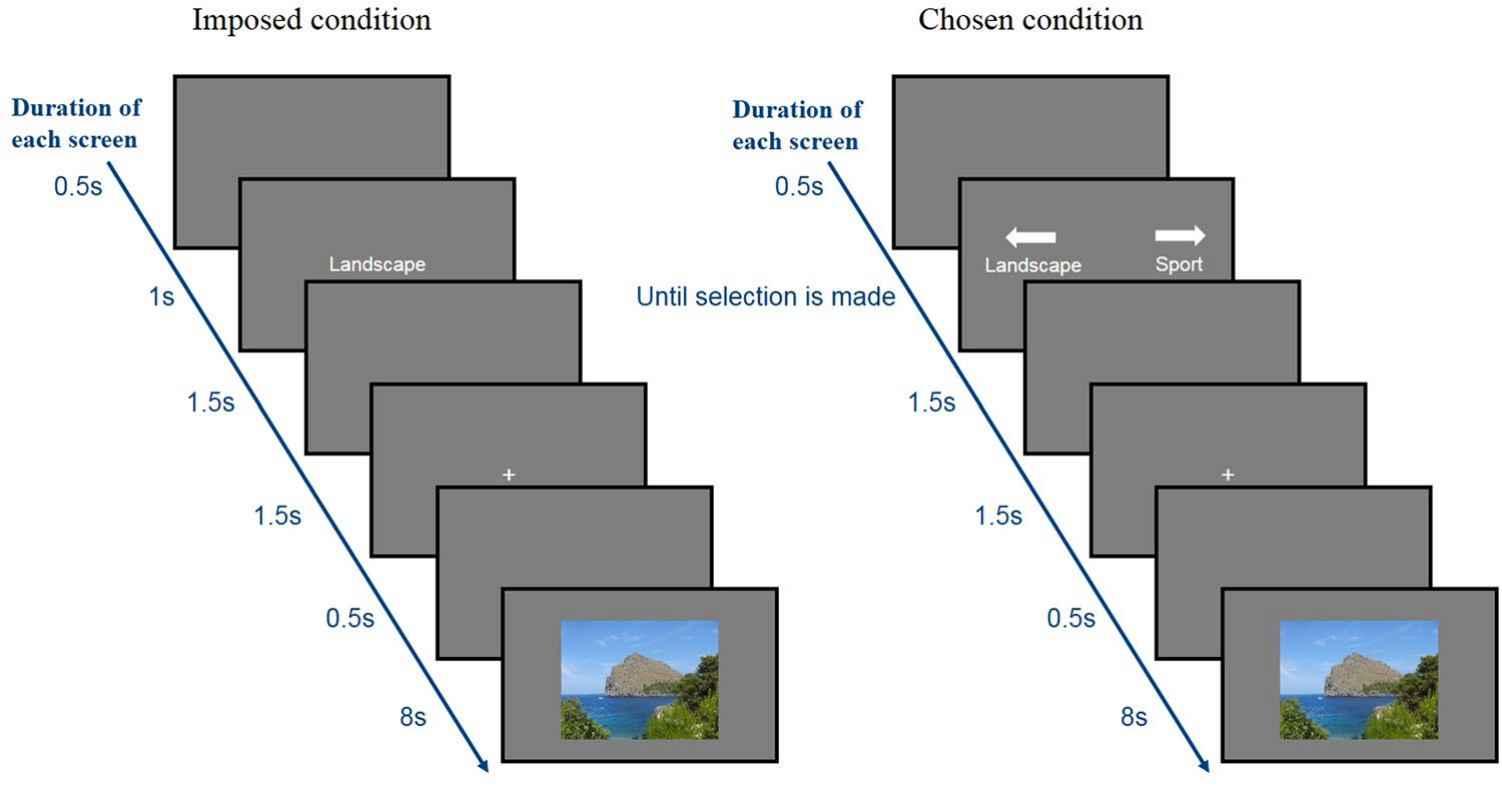
Unfolding of the Imposed condition (left side) and of the Chosen condition (right side). The durations of each screen are indicated on the side of the blue arrows.

This study had a within-subject design, assuming that participants would react similarly to the same images, permitting to tackle the sole effect of choice. All participants saw blocks of pictures of the Imposed and the Chosen conditions. Each participant went through 10 blocks of trials, each separated by a screen through which participants could progress at their own pace, allowing them to take breaks when needed. Four blocks were under the Imposed condition and six were under the Chosen condition, presented in a semi-randomized order with no more than two consecutive blocks of the same condition. Imposed blocks were composed of 24 trials (12 positives and 12 negatives, each with 3 images of each content type). Chosen blocks were generally composed of 16 trials (8 positives and 8 negatives, each with 2 images of each content type). The last chosen block differed in the number of trials. This is due to the pairing procedure, the program exiting the last Chosen block once no more option for pairing balanced contents and unseen images in Chosen condition was available. Number of pictures differed between blocks in the two conditions so as to match block durations, Chosen condition taking more time than imposed lists of images. Figure 1 shows the unfolding of the two types of conditions.

Images were randomly chosen among their content type but only presented maximally once per participant per condition. On average, participants performed 188 trials, 96 Imposed and 92 Chosen (three blocks of 16 trials and the last one consisting of 12 trials on average). Only the images of the Imposed condition that were also seen in the Chosen condition were retained for analysis in order to have a perfect match of intrinsic emotional and perceptive content in the two conditions. This led to the analyses of 80 trials in the negative context (40 Chosen, 40 Imposed), and 90 (45 Chosen, 45 Imposed) in the positive context. The condition orders for each image pair were randomized across all the participants, thus cancelling out the contribution of habituation effects in our comparisons. After the computer session, which lasted about 55 min, sensors were removed and participants were fully debriefed.

All participants gave informed consent for participating in the study. The procedure was reviewed and authorized by institutional and regional ethical committee (CER-VD, protocol 2015-00071), in accordance with the current national legal requirements (Ordinance on Human Research) and the latest version of the declaration of Helsinki.

### Data Reduction

All the recordings were treated with Acknowledge 4.4 (Biopac Systems). Some channels were band-pass filtered to increase signal to noise ratios (20–500 Hz for EMG, 0.5–35 Hz for ECG, 0.15–7 Hz for finger pulse, 0.05–1 Hz for respiration). Channels were then manually scanned for movements or electric interferences, which were corrected via signal interpolation. To assess the temporal dynamic of emotional response unfolding, the continuous parameters were segmented into 16 epochs of 0.5 s each. Note that parameters were first calculated on all experiments before being segmented, therefore reducing the artifact of time segmentation limits. In addition to the picture presentation period (8 s of picture viewing), a baseline of 3.5 s was calculated for each trial and each parameter. This period spanned from 3.5 s before the picture presentation to the time of the picture onset and represented for each trial for all conditions a blank screen and the fixation cross (see Fig. 1).

#### Emotional experience

Ratings were exported to obtain mean values for each epoch. Calculated baseline served as 0 point to calculate emotion intensity. Rating data were transformed into an emotion intensity scale. Output was extracted in percentage, representing how far the slider was pushed between its 0 point (baseline) and its extreme value on either side. Data for each of the valence side go from 0 = absence of added emotional feeling to 100 = extreme emotion intensity.

#### Emotion-expressive behaviour

EMG signals were rectified and smoothed (5 Hz) before being averaged for each epoch. Given the high variability in contraction capacity in each individual, each EMG time frame value was then expressed as the percentage of contraction with respect to the corresponding trial baseline level (voltage recorded for a given time frame/voltage recorded during baseline * 100)^59,60^.

#### Autonomic and somatic responses

Heart rate was calculated from the ECG channel by transforming the inter-beat interval (obtained by the calculation of the duration between successive R waves). Skin conductance level, pulse amplitude, pulse transit time (i.e., the time interval between the R wave of the ECG and the upstroke of the peripheral pulse at the finger site), and temperature were exported as mean values for each epoch. A Finger Pulse Composite was then created by averaging the z-scores of pulse amplitude and transit time, similarly to a procedure in a previous study^37^, and motivated by the positive correlation found between the two parameters (*r* = 0.30, *p* = 0.017). Respiratory rate and respiratory amplitude were calculated for each epoch. The respiratory rate was obtained by converting the duration of the cycle intervals into a number of cycles per minute (c/min). The respiratory amplitude was interpolated by using the difference in volts between the point of maximum inspiration and the point of maximum expiration. Given the high correlations between thoracic and abdominal respiratory rates (*r* = 0.55, *p* < 0.001), these parameters were averaged. Similarly, thoracic and abdominal respiratory amplitudes were averaged for analyses. Leg movements were calculated by exporting for each epoch the maximum of the sum of the rectified and smoothed (5 Hz) signal of the three channels (axis X, Y, Z) for obtaining general activity, irrespective of the direction. All these response channel data were calculated as the change in activity with respect to each trial baseline.

### Data Analyses

For each participant and each parameter, each time epoch of negative trials was averaged, once for the Imposed condition and once for the Chosen condition. Similarly, each time epoch of positive trials was averaged for each condition (Imposed vs. Chosen). ANOVAs were used to contrast our conditions with two within-factors: Regulation (2 levels: Imposed condition vs. Chosen condition) and Time (16 epochs). Since contrasting positive and negative trials was not part of our research question and since previous research has shown different emotion^61–66^ and emotion regulation patterns^67–69^ for positive and negative responses, separate ANOVAs were performed for negative and positive trials. For this study, two effects were of interest: (i) the main effect of Regulation, for evaluating the general effect of choosing as compared to the Imposed condition, and (ii) the interaction effects Regulation x Time, particularly interesting to evaluate the *temporal dynamics* of choice effects. Greenhouse-Geisser corrections have been applied where the assumption of sphericity was violated, and corrected degrees of freedom have been reported in such cases. Effect sizes are reported using partial eta square (η_p_^2^) and confidence intervals are reported where appropriate. *P*-values for interaction effect investigations were corrected for multiple comparisons with the Holm-Bonferroni criterion. Threshold for significance for all effects was set to .05 (two-tailed). All matrices are available from the last author for research-related follow-ups.

## Results

For each condition, the average values, Standard Error of the Mean (SEM), and 95% confidence intervals on the whole trial duration for each parameter are reported in Table 1 for negative trials and in Table 2 for positive trials.

**Table 1:** Mean, SEM, and 95% CI of Experiential, Expressive, and Autonomic and Somatic Responses to Negative Stimulations for Imposed and Chosen conditions.

	Imposed			Chosen		
	Mean	SEM	Confidence Interval (95%)	Mean	SEM	Confidence Interval (95%)
**Emotion experience**						
Rating (/100)	40.47	2.49	[35.50, 45.45]	38.17	2.30	[33.58, 42.75]
**Emotion-expressive behaviour**						
Corrugator (%)	130.84	7.1	[116.66, 145.02]	138.59	8.21	[121.18, 154.01]
**Autonomic and somatic responses**						
Δ HR (bpm)	−1.78	0.17	[−2.12, −1.44]	−1.89	0.17	[−2.23, −1.55]
Δ SCL (microS)	−0.02	0.014	[−0.047, 0.007]	−0.022	0.017	[−0.057, 0.013]
Δ FT (°C)	0.004	0.001	[0.001, 0.006]	0.003	0.001	[0.001, 0.006]
Δ FP (z-score)	0.094	0.065	[−0.035, 0.223]	−0.127	0.082	[−0.29, 0.037]
Δ RR (c/min)	0.11	0.047	[0.016, 0.204]	0.01	0.05	[−0.089, 0.110]
Δ RA (mV)	4.08	6.44	[−8.78, 16.93]	−7.47	8.52	[−24.49, 9.56]
Δ Leg movement (g E-4)	−7.71	3.9	[−15.5, 0.075]	−7.10	2.69	[−12.5, −1.73]

*Note*. The experience scale goes from 0 (*no emotion*) to +100 (*very negative*). Expressivity is expressed as percentage of baseline level. All other parameters are differences from baseline level. HR = Heart Rate, SCL = Skin Conductance Level, FT = Finger Temperature, FP = Finger Pulse Composite, RR = Respiratory Rate, RA = Respiratory Amplitude.

**Table 2:** Mean, SEM, and 95% CI of Experiential, Expressive, and Autonomic and Somatic Responses to Positive Stimulations for Imposed and Chosen conditions.

	Imposed			Chosen		
	Mean	SEM	Confidence Interval (95%)	Mean	SEM	Confidence Interval (95%)
**Emotion experience**						
Rating (/100)	44.15	1.84	[40.47, 47.83]	43.46	1.73	[40.01, 46.92]
**Emotion-expressive behaviour**						
Zygomaticus (%)	147.04	5.91	[135.23, 158.85]	144.73	5.47	[133.80, 155.67]
Orbicularis (%)	132.16	4.54	[123.09, 141.23]	132.74	5.05	[122.65, 142.82]
**Autonomic and somatic responses**						
Δ HR (bpm)	−1.07	0.16	[−1.38, −0.75]	−1.48	0.18	[−1.84, −1.13]
Δ SCL (microS)	−0.065	0.014	[−0.092, −0.037]	−0.034	0.012	[−0.058, −0.01]
Δ FT (°C)	0.002	0.001	[0.000, 0.005]	0.004	0.001	[0.002, 0.007]
Δ FP (z-score)	0.098	0.055	[−0.012, 0.208]	−0.06	0.06	[−0.179, 0.059]
Δ RR (c/min)	0.074	0.054	[−0.034, 0.182]	0.046	0.052	[−0.057, 0.149]
Δ RA (mV)	−4.43	7.82	[−20.05, 11.20]	5.15	7.36	[−9.56, 19.86]
Δ Leg movement (g E-4)	−7.06	3.81	[−14.6, 0.56]	−8.02	3.19	[−14.4, −1.64]

*Note*. The experience scale goes from 0 (*no emotion*) to +100 (*very positive*). Expressivity is expressed as percentage of baseline level. All other parameters are differences with baseline level. HR = Heart Rate, SCL = Skin Conductance Level, FT = Finger Temperature, FP = Finger Pulse Composite, RR = Respiratory Rate, RA = Respiratory Amplitude.

### Effects of choice on emotional experience

**Figure 2:**
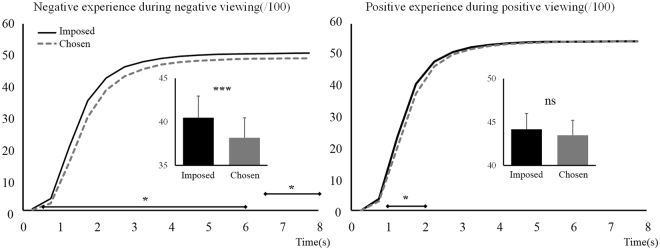
Emotional experience during negative (left) and positive (right) picture viewing. Scale is over 100. Imposed condition is represented with black continuous lines and Chosen condition with grey dashed lines. Main effects are represented embedded, error bars are SEM. Significant contrasts are given with diamond-ended lines along the time course. ns = non-significant, *p < 0.05 with Holm-Bonferroni corrections, ***p < 0.001.

During negative picture viewing, we observed a significant effect of Regulation, *F*_(1,64)_ = 23.01, *p* < 0.001, η_p_^2^ = 0.26, and a Regulation × Time interaction, *F*_(2,150)_ = 8.92, *p* < 0.001, η_p_^2^ = 0.12. Over the 8 s period of stimuli presentation, the Chosen condition induced reduced negative experience (38.17) as compared to the Imposed condition (40.47, see Table 1, and bar graph inset in Fig. 2, left side), which confirms our primary hypothesis. The line graph in Fig. 2 (left side) presents the temporal unfolding of negative experience emergence over the 8 seconds of picture presentation, with indications of time frames at which differentiation between conditions occurs.

During positive picture viewing, the effect of Regulation was not significant, *F*_(1,64)_ = 2.97, *p* = 0.09. A significant interaction Regulation × Time was however found, *F*_(2,134)_ = 7.85, *p* < 0.001, η_p_^2^ = 0.11, which however does not confirm our hypothesis of a positive experience enhancement. These results are illustrated in Fig. 2 (right side), with the same rationale as for negative viewing.

### Effects of choice on emotion-expressive behaviour

For negative trials, sixty-four observations were used for the electromyographic signal (EMG) analyses (1 participant signal was not recorded due to technical difficulties). Expressivity was assessed in this case with the *Corrugator Supercilii* EMG activity. The ANOVA showed a significant effect of Regulation, *F*_(1,63)_ = 6.61, *p* = 0.013, η_p_^2^ = 0.10. In the opposite direction to our expectations, this effect shows that observing a situation that has been chosen triggers more negative expressivity (139%) than when the same situation is imposed (131%, see also Table 1). The interaction between Time and Regulation was not significant, *F*_(5,320)_ = 0.86, *p* = 0.51.

For positive trials, expressivity was assessed with the *Zygomaticus Major* and the *Orbicularis Oculi* EMG activity for 64 participants (each time one participant signal was not recorded due to technical difficulties). Contrary to our expectation, *Situation selection* did not significantly impact positive expressivity. For the zygomatic region, results show that neither the Regulation effect, *F*_(1,63)_ = 0.66, *p* = 0.42, nor the interaction Regulation × Time, *F*_(4,264)_ = 1.39, *p* = 0.24, were significant. Similarly, for the orbicular region, neither the Regulation effect, *F*_(1,63)_ = 0.06, *p* = .81, nor the interaction Regulation × Time, *F*_(4,235)_ = 1.66, *p* = 0.17, were significant.

### Effects of choice on autonomic and somatic responses

#### Heart Rate

**Figure 3:**
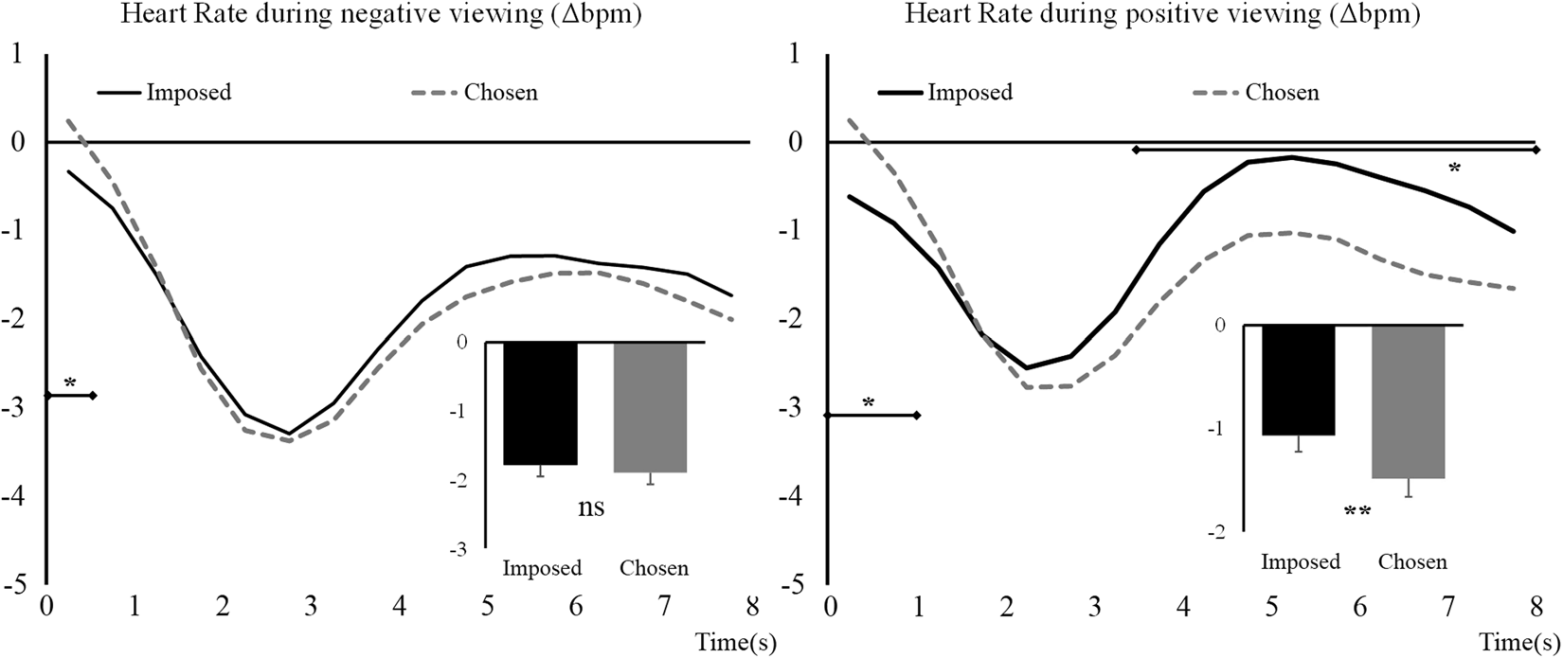
Heart rate difference with baseline during negative (left) and positive (right) picture viewing. Imposed condition is represented with black continuous lines and Chosen condition with grey dashed lines. Main effects are represented embedded, error bars are SEM. Significant contrasts are given with diamond-ended lines along the time course. Bpm = beat per minute, ns = non-significant, *p < 0.05 with Holm-Bonferroni corrections, **p < 0.01.

For negative trials, analyses showed a non-significant effect of Regulation, *F*_(1,64)_ = 0.36, *p* = 0.55, but a significant Regulation × Time interaction, *F*_(4,271)_ = 2.65, *p* = 0.031, η_p_^2^ = 0.04. These results are illustrated in Fig. 3 (left side) and show that the Imposed condition (as compared to the Chosen condition) triggers a faster decrease in heart rate in the first half second of the viewing. Interestingly, and contrary to our hypothesis for this parameter, orienting phase was not impacted by *Situation selection*.

For positive trials, the ANOVA showed an effect of Regulation, *F*_(1,64)_ = 7.28, *p* = 0.01, η_p_^2^ = 0.10 and a Regulation × Time interaction, *F*_(4,269)_ = 17.37, *p* < 0.001, η_p_^2^ = 0.21. These effects are illustrated in Fig. 3 (right side). Over the 8 s period considered, and contrary to our hypothesis, participants showed an overall stronger decrease in heart rate with respect to baseline in the Chosen condition, as compared to the Imposed condition (see Table 2). The data dynamic shows that the Imposed condition (as compared to the Chosen condition) triggers a faster decrease in heart rate in the first second of viewing. More interesting, Chosen condition induces a stronger decrease of heart rate as compared to baseline in the last 4.5 s of the viewing. Thus, at the end of the viewing period, heart rate for positive viewing has decreased more in the Chosen condition than in the Imposed.

#### Skin conductance level

**Figure 4:**
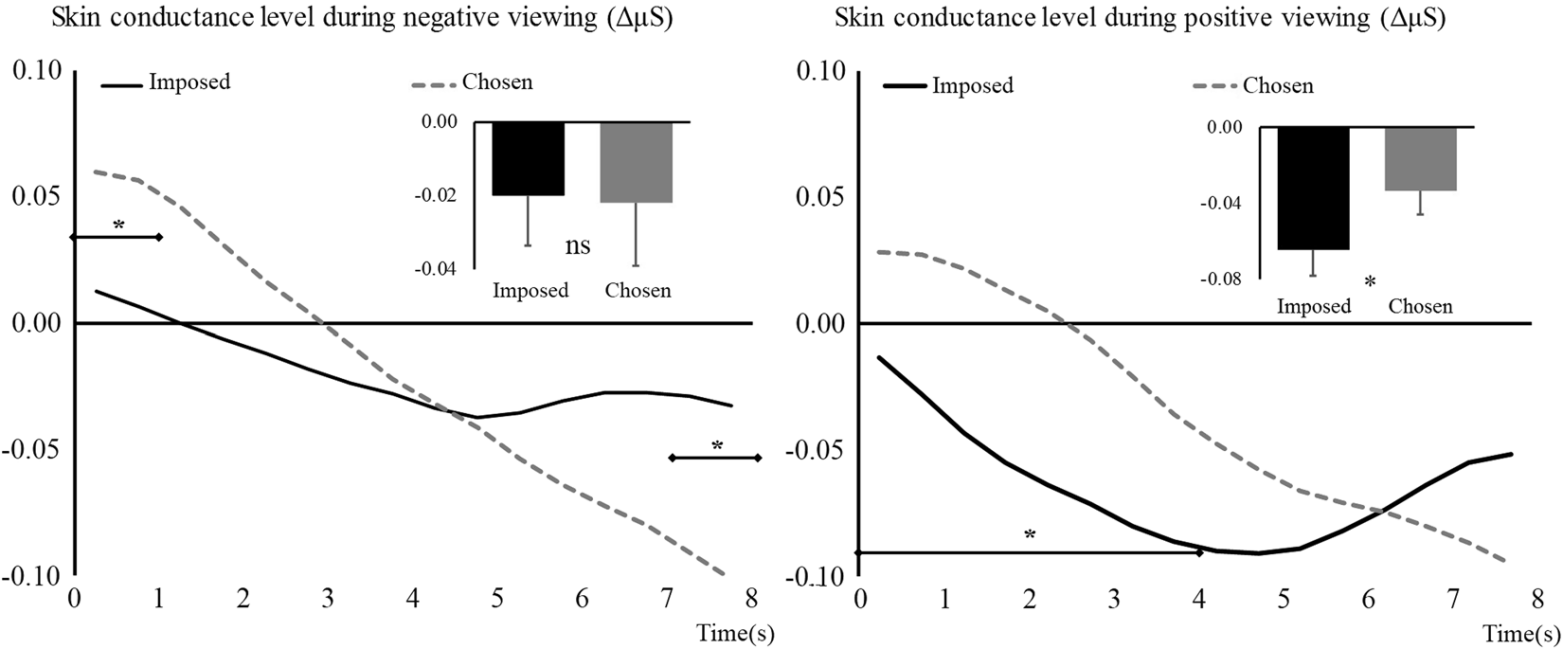
Skin conductance level difference with baseline during negative (left) and positive (right) picture viewing. Imposed condition is represented with black continuous lines and Chosen condition with grey dashed lines. Main effects are represented embedded, error bars are SEM. Significant contrasts are given with diamond-ended lines along the time course. ns = non-significant, *p < 0.05 with Holm-Bonferroni corrections.

Fifty one observations were used for the skin conductance analyses. Fourteen participants were excluded as they were non-responders for this measure (also known as labile), showing low skin conductance level, fast habituation to stimuli presentation, as well as steady tonic decrease over the course of the study^70–72^. For negative trials, analyses did not show a significant effect of Regulation, *F*_(1,50)_ = 0.02, *p* = 0.89. However, a significant interaction Regulation × Time was found, *F*_(2,80)_ = 8.86, *p* = 0.001, η_p_^2^ = 0.15. These results are illustrated in Fig. 4 (left side). They show a distinction between early and late viewing, characterized by a stronger increase in skin conductance level in the Chosen condition (as compared to the Imposed one) in the first second of the viewing. Conversely, in the last second, this condition shows, as hypothesized, a stronger decrease in skin conductance, as compared to the Imposed condition.

For positive trials, a significant effect of Regulation, *F*_(1,50)_ = 4.41, *p* = 0.04, η_p_^2^ = 0.08, and a significant interaction Regulation × Time, *F*_(2,119)_ = 16.64, *p* < 0.001, η_p_^2^ = 0.25, were found. The main effect shows that, when chosen, the images trigger a stronger decrease in skin conductance level (−0.065 μS), as compared to when images are imposed (−0.034 μS, see also Table 1). These results are illustrated in Fig. 4 (right side). Contrary to negative viewing, differences between our conditions were observed only during one period (the first four seconds), during which the increase in skin conductance in the Chosen condition was significantly different from the decrease observed in the Imposed condition.

#### Finger temperature

Finger temperature did not show sensitivity for our conditions, neither for negative trials (main effect of Regulation *F*_(1,64)_ = 0.04, *p* = 0.84, interaction *F*_(1,67)_ = 1.11, *p* = 0.30), nor for positive trials (main effect of Regulation *F*_(1,64)_ = 1.11, *p* = 0.30, interaction *F*_(1,67)_ = 0.14, *p* = 0.73).

#### Pulse

Sixty four observations were used for these analyses since the signal was not recorded for one participant due to technical difficulties. For negative trials, the finger pulse composite yielded a main effect of Regulation, *F*_(1,63)_ = 6.54, *p* = 0.01, η_p_^2^ = 0.09, but a non-significant interaction Regulation × Time, *F*_(3,217)_ = 1.24, *p* = 0.29. Contrary to expectations, the main effect shows that, when chosen, the images trigger a stronger decrease in composite (i.e., stronger decrease in pulse amplitude and transit time, −0.127 SD), as compared to when images are imposed (0.094 SD, see also Table 1).

For positive trials, the finger pulse composite yielded also a main effect of Regulation, *F*_(1,63)_ = 5.69, *p* = 0.02, η_p_^2^ = 0.08, but a non-significant interaction Regulation × Time, *F*_(4,269)_ = 1.48, *p* = 0.21. Again, the main effect shows that, when chosen, the image triggers a stronger decrease in composite (i.e., stronger decrease in pulse amplitude and transit time, -0.06 SD), as compared to when images are imposed, where an increase is observed (0.1 SD, see also Table 1).

#### Respiratory activity

**Figure 5:**
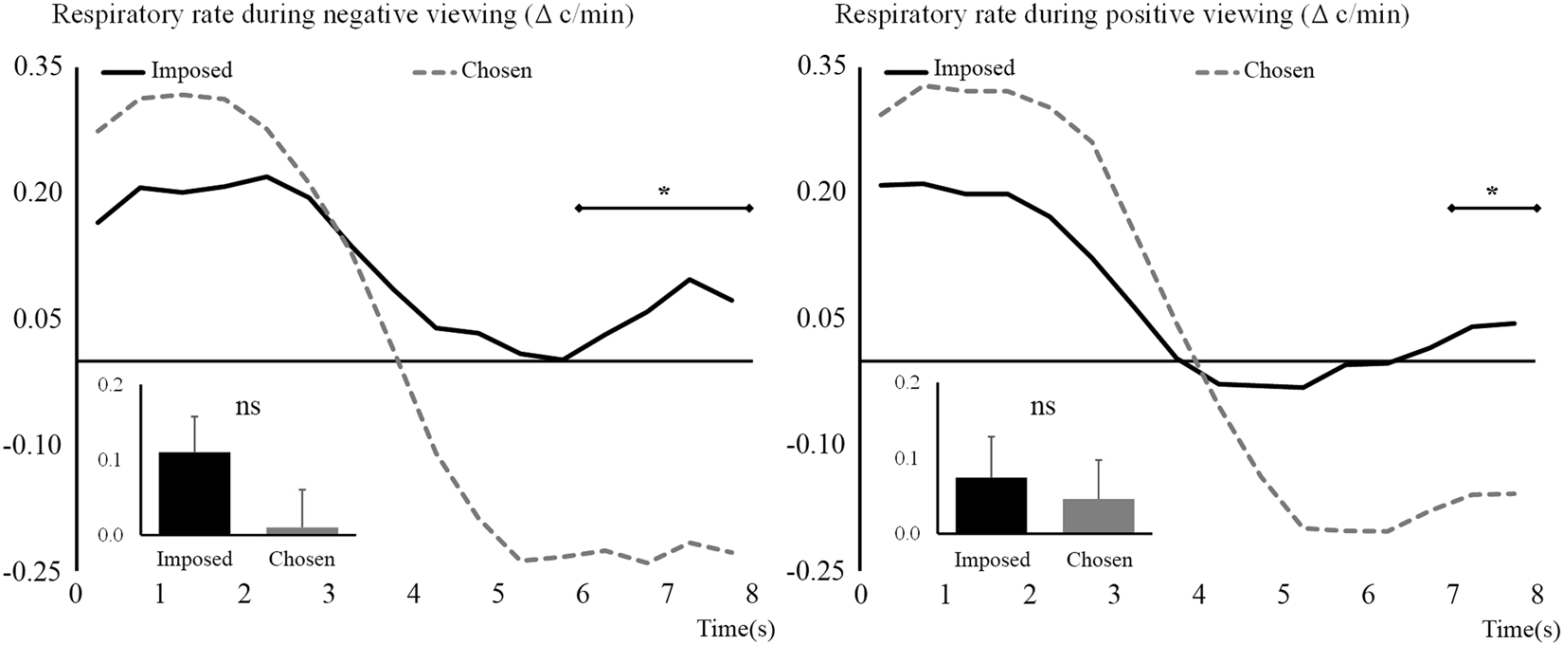
Respiratory rate difference with baseline during negative (left) and positive (right) picture viewing. Imposed condition is represented with black continuous lines and Chosen condition with grey dashed lines. Main effects are represented embedded, error bars are SEM. Significant contrasts are given with diamond-ended lines along the time course. ns = non-significant, *p < 0.05 with Holm-Bonferroni corrections.

Considering *respiratory rate*, the ANOVA performed on negative trials showed a non-significant effect of Regulation, *F*_(1,64)_ = 2.53, *p* = 0.12, but a significant Regulation × Time interaction, *F*_(2,156)_ = 9.26, *p* < 0.001, η_p_^2^ = 0.13. These results are illustrated in Fig. 5 (left side) and show that, after an initial increase in rate in both conditions, a decrease in respiratory rate occurs as predicted for the Chosen condition in the second half of the viewing period, distinguishing itself from the Imposed condition in the last two seconds. Similarly, analyses on respiratory rate for positive trials showed a non-significant effect of Regulation, *F*_(1,64)_ = 0.18, *p* = 0.68, but a significant Regulation × Time interaction, *F*_(2,144)_ = 8.43, *p* < 0.001, η_p_^2^ = 0.12. These results are illustrated in Fig. 5 (right side). The dynamic is the same as for the negative viewing, the decrease in respiratory rate being significant in the last second of the viewing period.

**Figure 6:**
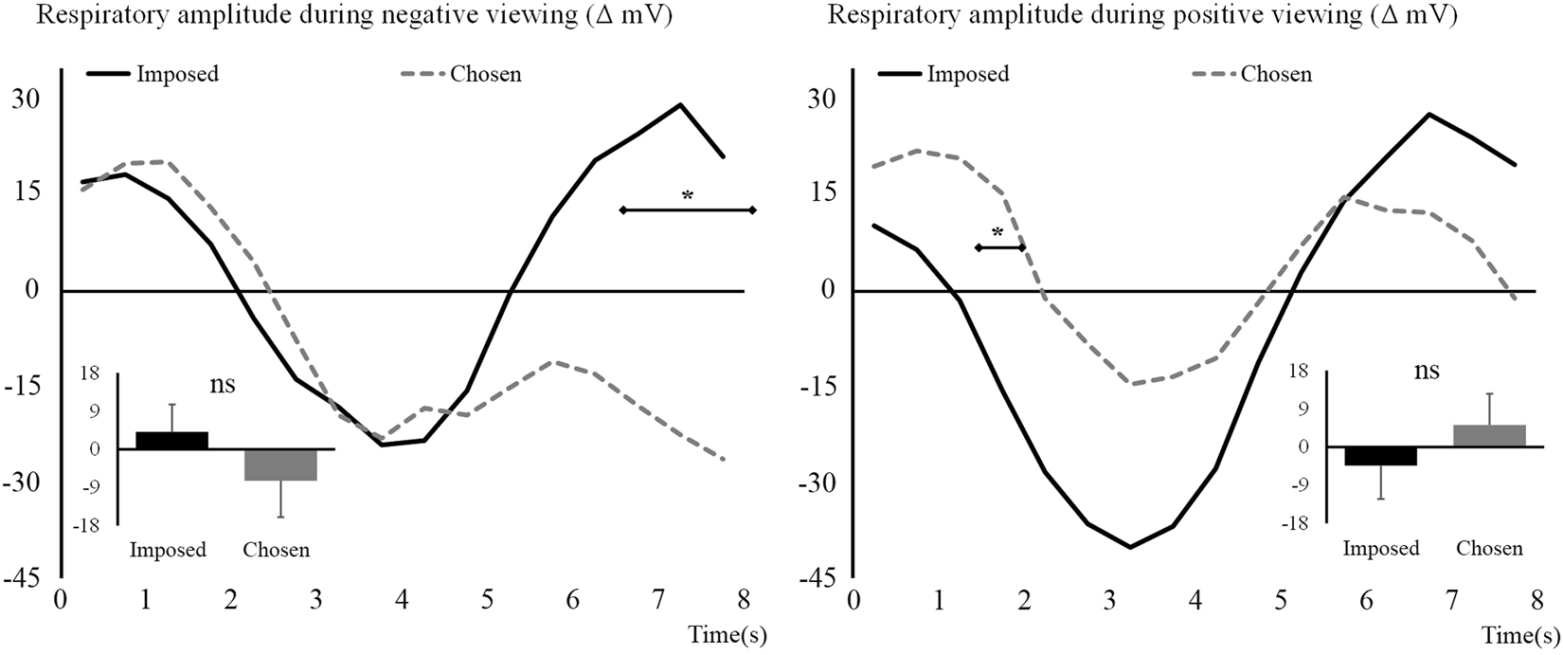
Respiratory amplitude difference with baseline during negative (left) and positive (right) viewing. Imposed condition is represented with black continuous lines and Chosen condition with grey dashed lines. Main effects are represented embedded, error bars are SEM. Significant contrasts are given with diamond-ended lines along the time course. ns = non-significant, *p < 0.05 with Holm-Bonferroni corrections.

Considering *respiratory amplitude*, the ANOVA performed on negative trials showed a non-significant effect of Regulation, *F*_(1,64)_ = 2.03, *p* = 0.16, but a significant Regulation × Time interaction, *F*_(3,163)_ = 4.37, *p* = 0.008, η_p_^2^ = 0.06. These results are illustrated in Fig. 6 (left side) and show, contrary to what was expected, an increase in amplitude in the Imposed condition in the last 1.5 s of the viewing period that is not observed in the Chosen condition. Similarly, analyses on respiratory amplitude for positive trials showed a non-significant effect of Regulation, *F*_(1,64)_ = 1.03, *p* = 0.32, but a significant Regulation × Time interaction, *F*_(2,142)_ = 4.31, *p* = 0.01, η_p_^2^ = 0.06. This time, however, the interaction results from a reduction in amplitude in the first half of the viewing period that is stronger for the Imposed than for the Chosen condition. These results are illustrated in Fig. 6 (right side).

#### Leg movement

Leg movement did not show sensitivity for our conditions, neither for negative trials (main effect of Regulation, *F*_(1,64)_ = 0.17, *p* = 0.68, interaction, *F*_(4,275)_ = 2.44, *p* = 0.052), nor for positive trials (main effect of Regulation, *F*_(1,64)_ = 0.90, *p* = 0.35, interaction, *F*_(4,260)_ = 1.23, *p* = 0.30).

## Discussion

Our goal in this study was to comprehensively examine for the first time the immediate emotional responses when we choose ourselves to be confronted with an emotional situation, as compared to when the same situation is imposed on us. Results of such a study permit to delineate if, in addition to choosing a situation that seems less arousing and/or more positive, the sole effect of choice (i.e., being allowed to select, at least to some extent, the situation) also has a regulatory impact by itself, altering emotion responses. This assessment was possible thanks to a specific design comparing reactions to the exact same stimulation material, with conditions only differing by the fact that images had been imposed or selected. One of the most striking results was that negative emotional exposure was less negative when chosen as compared to when imposed, as reflected by a reduction in the negative experience. Across our measures, the choice component of *Situation selection* impacted emotional responses at the experiential, physiological and somatic levels.

Regarding emotional experience, we hypothesized that, when confronted with a chosen situation, we should observe a decrease in negative experience and enhanced positive experience. Results indeed show that when choosing to live a negative situation, the associated negative experience is markedly reduced as compared to when the situation is not chosen. This observation is remarkable, because the lived situations were exactly the same. This means that, when given the choice, a same situation is lived with a less negative tone. This observation can be related to results from the empowerment literature. Here, researchers argued that choice may also be deleterious, e.g., by the too many options we regularly have^73^, negatively impacting happiness, optimism, self-esteem, life satisfaction, and learning^74–77^. On the other hand, freedom of choice regarding important life events is seen as an important aspect of self-motivation and sense of autonomy^32^ (see also the introduction section). Our study more specifically shows that, in the context of emotion situations, the empowerment gives to some extent the ability to live the situation as less negative, which is undeniably beneficial for short-term reactions to negative events. Our results showed that positive experience was generally not affected by choosing them, despite a short-lived slower rise in the experience onset. When choosing to live a positive emotion situation, the goal of *Situation selection* may be different than in negative viewing. Indeed, in the positive viewing, the benefit of the viewing could have lied in the reduction of pre-existing negative emotions, irrespective of the choice effect, hence resulting in an equally positive outcome of chosen and imposed positive stimulations.

For expressivity, we expected decreased negative and enhanced positive expressivity for the chosen condition. These hypotheses were not confirmed. At the postural level, lower limb activity showed no difference between conditions, for both negative and positive contexts. Facial expressivity was however amplified during the negative viewing. Together with the levels of negative experience, these results contradict the assumed coupling between expressivity and experience^78,79^. We could make the hypothesis that the decoupling is linked to the communication function of expressivity. As they know they are being observed during the experiment (via the EMG), participants may need to communicate the expected emotions in a stronger fashion when they have expressly decided to watch a particular type of negative picture. This could be a signal so as to communicate to others that, despite the fact that they chose it, they know they are negative and that they are expected not to be insensitive. Similar mechanisms have been observed in pain, where expression of pain is enhanced in presence of others^80^. Hence, our data may witness, in addition to the expression of lived emotions, part of the social communicative aspect of expressivity, which are often unnoticed in laboratory studies.

Regarding physiological arousal, we expected for both valences a decrease of the cardiovascular orienting effect and skin conductance level. The choice procedure in itself triggered an increase in heart rate and skin conductance level at the very beginning of the viewing period, indicating that performing a choice is arousing in itself. Chosen situations led, however, to decreased arousal at the end of the viewing period, as evidenced by a decrease in skin conductance level as compared to baseline. This drop was steeper than the one observed in the imposed condition. Similar patterns have been observed in low anxiety state^81^, suggesting a decrease in excretive reactions to arousing events. Contrary to expectation, however, the orienting phase (the first few seconds after image onset) did not differentiate between conditions. This result is to be related to the decrease of finger pulse composite during the Chosen condition, while imposed situations provoked an increase. In our data, a decrease of pulse composite is related to a decrease of transit time and amplitude, indicating increased activity of the sympathetic branch of the cardiovascular system. Such reactions are also shown in response to both positive and negative films^82^ and could be assimilated to engagement. In this particular setting, however, this effect seems weaker in the second part of the viewing period (see Supplementary Fig. S2), again speaking in favour of an arousal effect of the choice rather than a prolonged consequence thereof.

Regarding respiratory activity, chosen situations triggered during negative viewing a decrease in respiratory rate and amplitude at the end of the viewing period. This decrease corresponds to a slower and shallower respiration. Usually, negative emotions are associated with increased rate and amplitude of respiration, although rate and amplitude are not necessarily coupled in this manner^83^. In our study, we thus observe low arousing respiratory patterns, closer to calmness than the respiration observed in unregulated viewing. Similarly to negative viewing, respiratory rate showed a decrease during the Chosen condition at the end of the recording period for positive stimuli. The amplitude remained however similar between conditions at the end of the viewing period.

Altogether, these results seem to show that the choice mechanism involved in *Situation selection* helps regulating negative emotions by driving a decrease of experience, sympathetic excretive function, and respiration reactivity, while slightly enhancing expressivity and cardiovascular reactivity. Results show also that, contrary to other adaptive strategies, which also reduce positive experience^84^, the choice component of *Situation selection* is not deleterious, leaving positive experience and expressivity unaffected. With a decrease in heart rate and respiratory rate, the choice component of *Situation selection* may also partly reinforce the calming effect of positive situations.

This study is the first attempt to observe emotional consequences of the choice component of *Situation selection*. Future studies should modulate the choice given to the participants, which would deserve to be extended to a less limited and more ecological set of options. In this study, we also did not compare the reactions to the chosen situations vs. to the ignored situations. Rather, we compared reactions to identical situations, whether they are selected or imposed. This comparison gave us the unique ability to see the consequences of choice, irrespective of final situation. Past results have concentrated on the arousal and valence aspects of the stimulation, which serve as trigger for the choice (see e.g.^22^). It would be worthwhile now to investigate together the two mechanisms of *Situation selection*, targeting the interaction between the impact of choice and the arousal of the chosen situation. Moreover, now that we know that choice is already regulatory *per se*, it would be interesting to know which aspect of choice has such impact, for example by deciding whether it is the cognitive task involved, or the modification of the emotional timing due to individually-paced choice procedure. This would fully uncover *Situation selection* mechanisms. Another limitation relates to the consideration of broad emotion domains (negative vs. positive), concentrating on the differences between valence as brought up by dimensional theorists of affect^23,85,86^. This broad distinction had the advantage of limiting boundary problems between emotion categories linked to the frequent occurrence of mixed emotions^87^. Yet, this approach cannot inform on the efficiency of *Situation selection* for a particular emotion category with respect to another, as supposed by the supporters of discrete theories of affect^88–90^. Deeper investigations on the type of induced positive vs. negative affect could potentially explain the discrepant arousing effect we find regarding physiological reactivity to positive situations. Moreover, given that one theory cannot fully inform the emotional mechanisms into play^91,92^, other approaches may greatly improve our comprehension of *Situation selection* effects. We have chosen for this study to reduce the length of the more resource consuming block (Chosen condition) to equate the cognitive load and difficulties of each condition. The reverse (equating the length but not the load) could inform on the respective impact of this inherent difference between the conditions. Finally, the use of a between-subject design, despite lacking of control on individual differences in traits, appraisal and reactivity, may be additionally useful to decrease awareness and consequent differences in the participant responses.

To conclude, we investigated here one of the two mechanisms intervening in *Situation selection*, namely the effect of having the choice over an emotional situation we are about to experience. In addition, we offer a comprehensive description of the immediate emotional responses, at the experiential, expressive and physiological level to a *Situation selection* condition. Very few emotion regulation strategies succeed in reducing emotional experience. This seems however to be the case for *Situation selection*, particularly for negative situations. Despite a slight activation of the cardiovascular system, this decrease in negative experience, together with a lower arousal and respiratory pattern, turns *Situation selection* into a very promising efficient regulation strategy. It remains now to be shown if this effect reliably replicates with other kinds of choices, when confronted with different emotional stimuli, or in a non-student population. Nevertheless, choice seems to be a powerful tool for permitting to regulate negative emotions in an adaptive fashion, and could deserve to be implemented outside the laboratory. Finally, our results extend beyond emotion regulation as we show that choosing a situation improves the resultant experience, not only because we are able to anticipate a more positive situation, but primarily because we are just being given the choice.

## Electronic supplementary material

Supplementary Information

## References

[1] Bradley MM, Cuthbert BN, Lang PJ (1996). Picture media and emotion: Effects of a sustained affective context. Psychophysiology.

[2] Bradley, M. M., Greenwald, M. K. & Hamm, A. O. In *The* s*tru*ctur*e* of em*otion: Psychophysiological*, *cognitive*, *and clinical aspects* (eds N. Birbaumer & A. Öhman) 48–65 (Hogrefe & Huber, 1993).

[3] Frijda NH (1987). Emotion, cognitive structure, and action tendency. Cognition and Emotion.

[4] Bradley MM, Lang PJ (2000). Emotion and motivation. Handbook of psychophysiology.

[5] Levenson, R. W. In *The nature of emotion: Fundamental questions*. (eds P. Ekman & R. J. Davidson) 123–126 (Oxford University Press., 1994).

[6] Lang PJ (1995). The emotion probe: Studies of motivation and attention. American Psychologist.

[7] Gross, J. J. *Handbook of emotion regulation*. 2nd edn, (Guilford Press, 2014).

[8] Tomkins, S. In *Approaches to emotion* (eds K. Scherer & P. Ekman) 353–400 (Erlbaum, 1984).

[9] Gross JJ (1998). The emerging field of emotion regulation: An integrative review. Review of General Psychology.

[10] Gross JJ, John OP (2003). Individual differences in two emotion regulation processes: Implication for affect, relationships, and well-being. Journal of Personality and Social Psychology.

[11] Gross JJ, Muñoz RF (1995). Emotion regulation and mental health. Clinical Psychology: Science and Practice.

[12] Eisenberg N, Fabes RA, Guthrie IK, Reiser M (2000). Dispositional emotionality and regulation: Their role in predicting quality of social functioning. Journal of Personality and Social Psychology.

[13] Hayes SC, Wilson KG, Gifford EV, Follette VM, Strosahl K (1996). Experiential avoidance and behavioral disorders: A functional dimensional approach to diagnosis and treatment. Journal of Consulting and Clinical Psychology.

[14] Campbell-Sills, L. & Barlow, D. H. In *Handbook of emotion regulation* (ed James J. Gross) 542–559 (Guilford Press, 2007).

[15] Gross JJ (2001). Emotion regulation in adulthood: Timing is everything. Current Directions in Psychological Science.

[16] Gross, J. J. *Handbook of emotion regulation*. (Guilford Press, 2007).

[17] Webb TL, Miles E, Sheeran P (2012). Dealing with feeling: A meta-analysis of the effectiveness of strategies derived from the process model of emotion regulation. Psychological bulletin.

[18] Sheppes, G. & Gross, J. J. In *Handbook of Psychology*, *volume five: Personality and Social* Psychology. (eds H. A. Tennen & J. M. Suls) 391–406 (Wiley, 2012).

[19] Gross, J. J. & Thompson, R. A. In H*and*book of *em*otion *regulation* (ed J.J. Gross) 3–24 (Guilford Press, 2007).

[20] Livingstone KM, Isaacowitz DM (2015). Situation Selection and Modification for Emotion Regulation in Younger and Older Adults. Social Psychological and Personality Science.

[21] Rovenpor DR, Skogsberg NJ, Isaacowitz DM (2012). The choices we make: An examination of situation selection in younger and older adults. Psychology and Aging.

[22] Sands, M. & Isaacowitz, D. M. Situation selection across adulthood: the role of arousal. *Cognition and Emotion*, 1–8 (2016). 10.1080/02699931.2016.1152954 26983792

[23] Russell JA (1980). A circumplex model of affect. Journal of Personality and Social Psychology.

[24] Bradley MM, Lang PJ (1994). Measuring emotion: the self-assessment manikin and the semantic differential. Journal of behavior therapy and experimental psychiatry.

[25] De Leersnyder J, Boiger M, Mesquita B (2013). Cultural regulation of emotion: individual, relational, and structural sources. Frontiers in Psychology.

[26] Bresin K, Robinson MD (2015). You are what you see and choose: Agreeableness and situation selection. Journal of Personality.

[27] Leotti LA, Iyengar SS, Ochsner KN (2010). Born to choose: The origins and value of the need for control. Trends in cognitive sciences.

[28] Seligman, M. E. P. *Helplessness: on depression*, *development*, *and death*. (W.H. Freeman & Co, 1975).

[29] Abramson LY, Seligman ME, Teasdale JD (1978). Learned helplessness in humans: Critique and reformulation. Journal of abnormal psychology.

[30] Murayama, K., Izuma, K., Aoki, R. & Matsumoto, K. In *Recent Developments in Neuroscience Research on Human Motivation* 95–125 (Emerald Group Publishing Limited, 2016).

[31] Lemos A, Wulf G, Lewthwaite R, Chiviacowsky S (2017). Autonomy support enhances performance expectancies, positive affect, and motor learning. Psychology of Sport and Exercise.

[32] Deci EL, Ryan RM (2000). The “what” and “why” of goal pursuits: Human needs and the self-determination of behavior. Psychological Inquiry.

[33] Karsh N, Eitam B, Mark I, Higgins ET (2016). Bootstrapping agency: How control-relevant information affects motivation. Journal of Experimental Psychology: General.

[34] Stein, N. L., Trabasso, T. & Liwag, M. The representation and organization of emotional experience: Unfolding the emotion episode. (1993).

[35] Esslen M, Pascual-Marqui RD, Hell D, Kochi K, Lehmann D (2004). Brain areas and time course of emotional processing. NeuroImage.

[36] Dan-Glauser ES, Gross JJ (2015). The temporal dynamics of emotional acceptance: Experience, expression, and physiology. Biological Psychology.

[37] Dan-Glauser ES, Gross JJ (2011). The temporal dynamics of two response-focused forms of emotion regulation: Experiential, expressive, and autonomic consequences. Psychophysiology.

[38] Gross JJ (1998). Antecedent- and response-focused emotion regulation: Divergent consequences for experience, expression, and physiology. Journal of Personality and Social Psychology.

[39] Gross JJ (2002). Emotion regulation: Affective, cognitive, and social consequences. Psychophysiology.

[40] Ray RD, McRae K, Ochsner KN, Gross JJ (2010). Cognitive reappraisal of negative affect: converging evidence from EMG and self-report. Emotion.

[41] Krygier JR, Heathers JAJ, Gross JJ, Abbott M, Kemp AH (2014). Effect of emotion reappraisal on phasic cardiovascular responses to affective pictures. International Journal of Psychophysiology.

[42] Krygier JR, Heathers JAJ, Gross JJ, Abbott M, Kemp AH (2014). Emotion regulation and heart rate variability: Effect of reappraisal on cardiovascular responses to affective pictures. Psychophysiology.

[43] Codispoti M, Bradley MM, Lang PJ (2001). Affective reactions to briefly presented pictures. Psychophysiology.

[44] Lang PJ, Greenwald MK, Bradley MM, Hamm AO (1993). Looking at pictures: Affective, facial, visceral, and behavioral reactions. Psychophysiology.

[45] Bradley, M. M. & Lang, P. J. In Cogni*tiv*e ne*ur*oscie*nce of emotion* (eds R.D. Lane & L. Nadel) 242–276 (Oxford University Press, 2000).

[46] Cohen, J. *Statistical power analysis for the behavioral sciences*. 2nd edn, (Erlbaum, 1988).

[47] Ware JE, Kosinski M, Keller SD (1996). A 12-Item Short-Form Health Survey: construction of scales and preliminary tests of reliability and validity. Medical Care.

[48] Reuter-Lorenz P, Givis R, Moscovitch M (1983). Hemispheric specialization and the perception of emotion: evidence from right-handers and from inverted and non-inverted left-handers. Neuropsychologia.

[49] Bourne VJ (2008). Examining the relationship between degree of handedness and degree of cerebral lateralization for processing facial emotion. Neuropsychology.

[50] Oldfield RC (1971). The assessment and analysis of handedness: the Edinburgh inventory. Neuropsychologia.

[51] Dan-Glauser ES, Scherer KR (2011). The Geneva affective picture database (GAPED): A new 730-picture database focusing on valence and normative significance. Behavior Research Methods.

[52] Lang, P. J., Bradley, M. M. & Cuthbert, B. N. International Affective Picture System (IAPS): Instruction manual and affective ratings. (University of Florida., Technical Report A-4, Center for Research inPsychophysiology, 1999).

[53] Kring AM, Gordon AH (1998). Sex differences in emotion: expression, experience, and physiology. Journal of personality and social psychology.

[54] Matsumoto D, Nezlek JB, Koopmann B (2007). Evidence for universality in phenomenological emotion response system coherence. Emotion.

[55] Mauss IB, Levenson RW, McCarter L, Wilhelm FH, Gross J (2005). The tie that binds? Coherence among emotion experience, behavior, and physiology. Emotion.

[56] Larsen JT, Norris CJ, Cacioppo JT (2003). Effects of positive and negative affect on electromyographic activity over zygomaticus major and corrugator supercilii. Psychophysiology.

[57] Frank MG, Ekman P, Friesen WV (1993). Behavioral markers and recognizability of the smile of enjoyment. Journal of personality and social psychology.

[58] Fridlund AJ, Cacioppo JT (1986). Guidelines for human electromyographic research. Psychophysiology.

[59] Van Boxtel, A. In *Proceedings of measuring behavior*. (eds A. J. Spink *et al*.) 104–108 (Noldus Information Technology).

[60] de Wied M, van Boxtel A, Zaalberg R, Goudena PP, Matthys W (2006). Facial EMG responses to dynamic emotional facial expressions in boys with disruptive behavior disorders. Journal of Psychiatric research.

[61] Lang, P. J., Bradley, M. M. & Cuthbert, B. N. In *Attention and orienting: Sensory and motivational processes* Vol. 97–136 (eds P. J. Lang, R. F. Simons, & M. T. Balaban) (Erlbaum Associates, 1997).

[62] Palomba D, Angrilli A, Mini A (1997). Visual evoked potentials, heart rate responses and memory to emotional pictorial stimuli. International journal of psychophysiology.

[63] VanOyen Witliet C, Vrana SR (1995). Psychophysiological responses as indices of affective dimensions. Psychophysiology.

[64] Kensinger EA, Schacter DL (2006). Processing emotional pictures and words: effects of valence and arousal. Cognitive, Affective, & Behavioral Neuroscience.

[65] Dolcos F, LaBar KS, Cabeza R (2004). Dissociable effects of arousal and valence on prefrontal activity indexing emotional evaluation and subsequent memory: an event-related fMRI study. Neuroimage.

[66] Winton WM, Putnam LE, Krauss RM (1984). Facial and autonomic manifestations of the dimensional structure of emotion. Journal of Experimental Social Psychology.

[67] Hubert W, de Jong-Meyer R (1991). Autonomic, neuroendocrine, and subjective response to emotion-inducing film stimuli. International Journal of Psychophysiology.

[68] Mak AK, Hu Z-G, Zhang JX, Xiao Z-W, Lee TM (2009). Neural correlates of regulation of positive and negative emotions: an fMRI study. Neuroscience letters.

[69] Kim SH, Hamann S (2007). Neural correlates of positive and negative emotion regulation. Journal of cognitive neuroscience.

[70] Mundy-Castle A, McKiever B (1953). The psychophysiological significance of the galvanic skin response. Journal of Experimental Psychology.

[71] Dawson ME, Nuechterlein KH (1984). Psychophysiological dysfunctions in the developmental course of schizophrenic disorders. Schizophrenia Bulletin.

[72] Gruzelier JH (2009). Clinical attributes of schizophrenic skin conductance responders and non-responders. Psychological Medicine.

[73] Chua, R. Y. J. & Yengar, S. S. In *Research in Or*ganiz*ational Behavior* Vol. 27 *Research in Organizational Behavior* (ed B. M. Staw) 41–79 (Jai-Elsevier Science Inc, 2006).

[74] Markus HR, Schwartz B (2010). Does choice mean freedom and well-being?. Journal of Consumer Research.

[75] Schwartz B (2009). Incentives, choice, education and well-being. Oxford Review of Education.

[76] Roets A, Schwartz B, Guan Y (2012). The tyranny of choice: a cross-cultural investigation of maximizing-satisficing effects on well-being. Judgment and Decision Making.

[77] Schwartz B (2002). Maximizing versus satisficing: happiness is a matter of choice. Journal of Personality and Social Psychology.

[78] Buck R (1980). Nonverbal behavior and the theory of emotion - The facial feedback hypothesis. Journal of Personality and Social Psychology.

[79] McIntosh DN (1996). Facial feedback hypotheses: Evidence, implications, and directions. Motivation and Emotion.

[80] Williams ACdC (2002). Facial expression of pain, empathy, evolution, and social learning. Behavioral and brain sciences.

[81] Chattopadhyay P, Cooke E, Toone B, Lader MH (1980). Habituation of physiological responses in anxiety. Biological Psychiatry.

[82] Gross JJ, Levenson RW (1997). Hiding feelings: The acute effects of inhibiting negative and positive emotion. Journal of Abnormal Psychology.

[83] Boiten FA, Frijda NH, Wientjes CJE (1994). Emotions and respiratory patterns: Review and critical analysis. International journal of psychophysiology.

[84] Kanske P, Heissler J, Schönfelder S, Bongers A, Wessa M (2011). How to regulate emotion? Neural networks for reappraisal and distraction. Cerebral Cortex.

[85] Posner J, Russell JA, Peterson BS (2005). The circumplex model of affect: An integrative approach to affective neuroscience, cognitive development, and psychopathology. Development and psychopathology.

[86] Barrett LF, Mesquita B, Ochsner KN, Gross J (2007). J. In *Annual Review of Psychology*. Annual Review of Psychology.

[87] Ellsworth, P. C. & Scherer, K. R. In *Handbook of Af*fecti*ve Sciences* (eds R.J. Davidson, K.R. Scherer, & H.H. Goldsmith) 572–595 (Oxford University Press, 2003).

[88] Ekman P (1992). An argument for basic emotions. Cognition and emotion.

[89] Roseman IJ, Wiest C, Swartz TS (1994). Phenomenology, behaviors, and goals differentiate discrete emotions. Journal of Personality and Social Psychology.

[90] Roseman IJ, Spindel MS, Jose PE (1990). Appraisal of emotion-eliciting events - Testing a theory of discrete emotions. Journal of Personality and Social Psychology.

[91] Barrett LF (1998). Discrete emotions or dimensions? The role of valence focus and arousal focus. Cogn. Emot..

[92] Christie IC, Friedman BH (2004). Autonomic specificity of discrete emotion and dimensions of affective space: a multivariate approach. International journal of psychophysiology.

