# Supplementary material for: The regulatory effect of choice in Situation Selection reduces experiential, exocrine and respiratory arousal for negative emotional stimulations
Source: Sci Rep. 2017 Oct 3;7:12626. doi: 10.1038/s41598-017-12626-7 (PMC5626686; doi:10.1038/s41598-017-12626-7)
Supplement: Supplementary file 1 — Supplementary Information [file 41598_2017_12626_MOESM1_ESM.pdf]

The regulatory effect of choice in Situation Selection reduces experiential, exocrine and respiratory arousal for negative emotional stimulations

Simon Thuillard and Elise S. Dan-Glauser*
University of Lausanne, Switzerland

Supplementary Note

Supplementary Figure S1

Supplementary Figure S2

Supplementary Note

Presented below is a list of other psychological parameters measured in Session 1 (see Method section). Indication of the instrument names and their reference are also included. These questionnaires served for another study and were not included in the data analyses of the present study.

- Current emotion state, measured with the PANAS[1]
- Standard emotion behavior, measured with the BEQ[2]
- Standard emotion regulation behavior, measured with the ERQ[3], the ERP-R[4], and the CERQ[4-6]
- Difficulties in emotion inner perception, measured with the TAS[7,8]
- Difficulties in applying emotion regulation, measured with the DERS[9]
- Social desirability bias, measured with the MCSDS[10]

1 Watson, D., Clark, A. L. & Tellengen, D. Development and validation of brief measure of positive and negative affect : The PANAS scales. *Journal of Personality and Social Psychology* **54**, 1063-1070 (1988).

2 Gross, J. J. & John, O. P. Revealing feelings: Facets of emotional expressivity in self-reports, peer ratings, and behavior. *Journal of Personality and Social Psychology* **72**, 435-448, doi:10.1037/0022-3514.72.2.435 (1997).

3 Gross, J. J. & John, O. P. Individual differences in two emotion regulation processes: implications for affect, relationships, and well-being. *Journal of personality and social psychology* **85**, 348 (2003).

4 Nelis, D., Quoidbach, J., Hansenne, M. & Mikolajczak, M. Measuring individual differences in emotion regulation: The Emotion Regulation Profile-Revised (ERP-R). *Psychologica Belgica* **51**, 49-91 (2011).

5 Gross, J. J. & John, O. P. Individual differences in two emotion regulation processes: Implication for affect, relationships, and well-being. *Journal of Personality and Social Psychology* **85**, 348-362 (2003).

6 Garnefski, N., Kraaij, V. & Spinhoven, P. Negative life events, cognitive emotion regulation and emotional problems. *Personality and Individual Differences* **30**, 1311-1327 (2001).

7 Bagby, R. M., Parker, J. D. A. & Taylor, G. J. The Twenty-Item Toronto Alexithymia Scale - I. Item selection and cross-validation of the factor structure. *Journal of Psychosomatic Research* **38**, 23-32 (1994).

8 Bagby, R. M., Taylor, G. J. & Parker, J. D. A. The Twenty-Item Toronto Alexithymia Scale - II. Convergent, discriminant, and concurrent validity. *Journal of Psychosomatic Research* **38**, 33-40 (1994).

9 Gratz, K. L. & Roemer, L. Multidimensional assessment of emotion regulation and dysregulation: development, factor structure, and initial validation of the Difficulties in Emotion Regulation Scale. *Journal of Psychopathology and Behavioral Assessment* **26**, 41-54 (2004).

10 Crowne, D. P. & Marlowe, D. A new scale of social desirability independent of psychopathology. *Journal of Consulting Psychology* **24**, 349-354 (1960).

Supplementary Figure S1 showing electromyographic measure outcomes

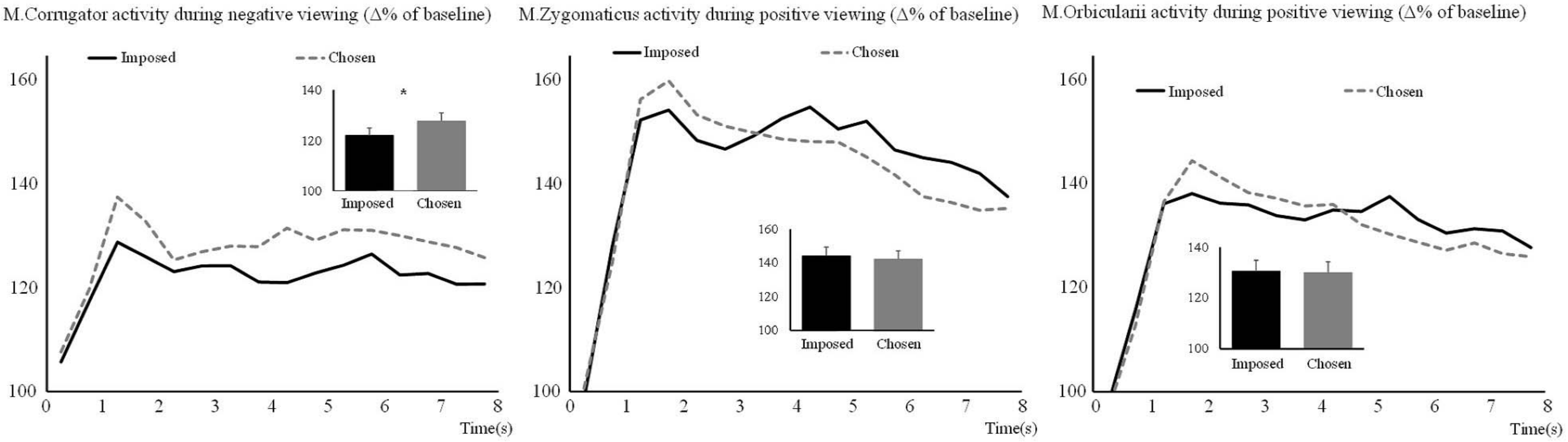

Figure S1: Muscular contractions as percentage of baseline level during negative (M. Corrugator muscle) and positive (middle, M. Zygomaticus Major, and right, M. Orbiculari Oculii) viewing. Imposed condition is represented with black continuous lines and Chosen condition with grey dashed lines. Main effects are represented embedded, error bars are SEM. *p<.05.

Supplementary Figure S2 showing temperature, pulse and somatic activity outcomes

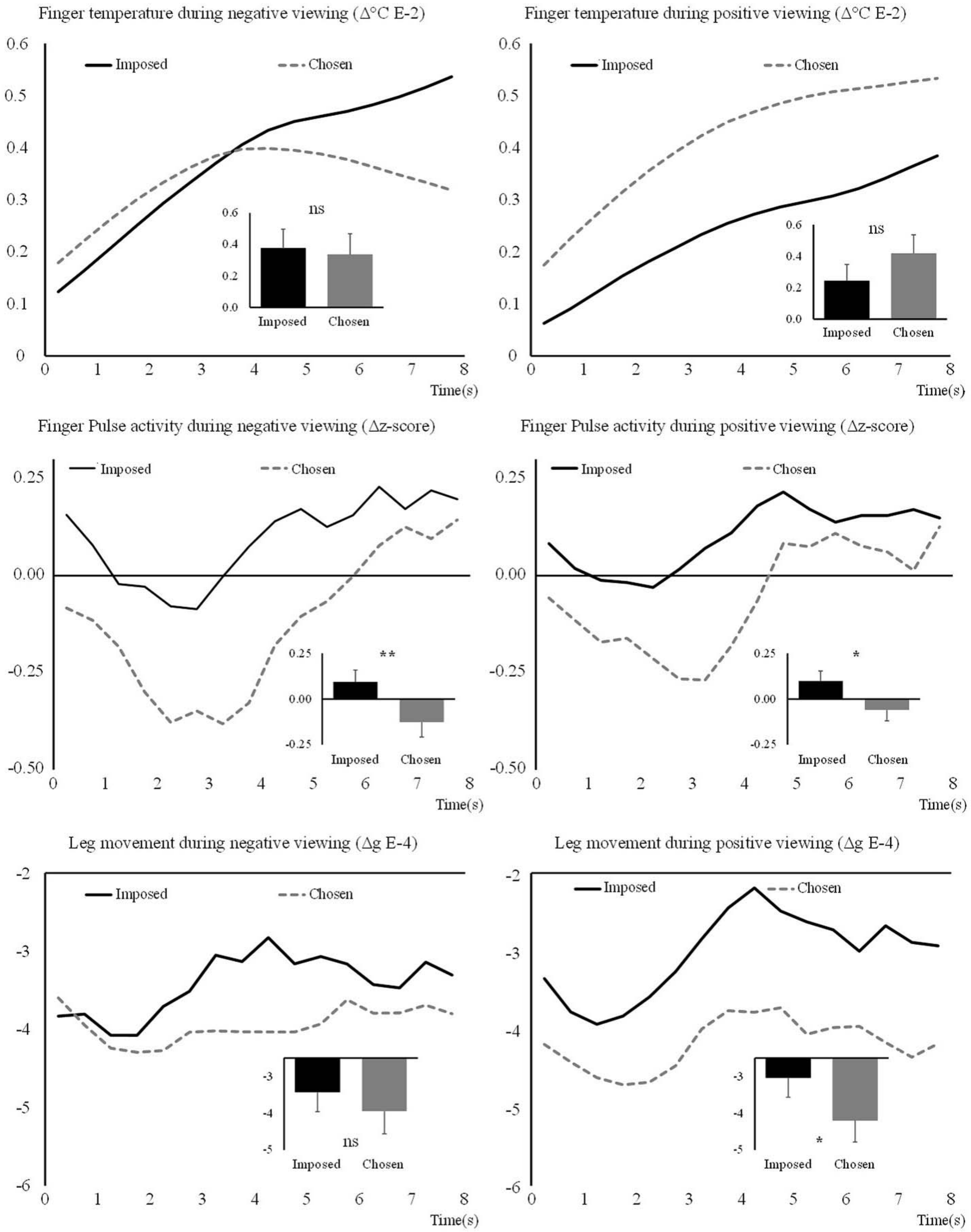

Figure S2: Finger temperature (top row), Finger Pulse (middle row), and Somatic activity (bottom row) as difference with baseline level during negative (left) and positive (right) viewing. Imposed condition is represented with black continuous lines and Chosen condition with grey dashed lines. Main effects are represented embedded, error bars are SEM. *p<.05, **<.01
